# Investigation of the Antioxidant and Cellular Toxicity Activities of Gold Nanoparticles Synthesized Using *Cichorium intybus* Extract on a Liver Cancer Cell Line

**DOI:** 10.5812/ijpr-159348

**Published:** 2025-04-14

**Authors:** Mohammad Mohammad-Alizadeh, Ahmad Asgharzadeh, Maryam Tatari

**Affiliations:** 1 Shirvan Branch, Islamic Azad University, Shirvan, Iran

**Keywords:** Gold Nanoparticles, Cytotoxicity, Antioxidant, *Cichorium intybus*

## Abstract

**Background:**

Liver cancer is increasing in different parts of the world and is the fourth leading cause of cancer death globally.

**Objectives:**

The present study aims to synthesize and analyze the characterization of gold nanoparticles (AuNPs) synthesized by *Cichorium intybus* extract and evaluate their antioxidant and cellular toxicity activity against liver cancer cells (HepG2).

**Methods:**

The synthesized AuNPs were characterized using X-ray diffraction (XRD), field emission scanning electron microscopy (FESEM), and fourier-transform infrared spectroscopy (FTIR). The antioxidant activity of the nanoparticles was assessed using the DPPH test, and their cytotoxicity activity was analyzed using the MTT method.

**Results:**

The results indicate that the AuNPs are crystalline materials with a particle size of less than 100 nm, with a mean particle size of 23.94 nm. The FTIR study reveals the presence of biochemical groups that act as reducing factors. The results demonstrate that antioxidant activity increases with concentration, with 87% inhibition of DPPH free radical scavenging observed at 250 µg/mL. Cell toxicity results in liver cancer cell lines (HepG2) demonstrated significant cytotoxicity in a time- and dose-dependent manner. The percentage of cell viability at a concentration of 1000 µg/ml after 24, 48, and 72 hours was determined to be 45%, 51%, and 22%, respectively.

**Conclusions:**

The present study revealed the simple cost-effective and environmentally friendly method that could be employed from food to pharmaceutical industries.

## 1. Background

Today, nanoscience has offered new opportunities for the treatment of various disorders. The eco-friendly, anti-cancer, antimicrobial, and antioxidant potential of nanoparticles has been extensively explored (1-5). Among nanomaterials, gold nanoparticles (AuNPs) have attracted significant attention due to their potential uses in medicinal and pharmacological studies (6, 7). The AuNPs are typically considered harmless materials to employ as medicine-carrying and antimicrobial resources (8). The AuNPs have garnered extensive attention in cancer treatment due to their low toxicity, great stability, simplicity of cellular uptake, and outstanding optical activities (9, 10).

The utilization of AuNPs in cancer treatment involves two main strategies. First, AuNPs can be employed as drug-delivery structures to enhance targeted distribution and efficiency (11). They can facilitate drug delivery in tumor tissues through the enhanced permeability and retention effect (12, 13). Treatments can be directly bound to the surface of AuNPs through various interactions (14, 15). The surface of AuNPs can be modified to simplify drug delivery.

In recent years, the antioxidant activities of metal and metal-oxide nanoparticles have been considered in several studies (16-18). Antioxidants play an essential role in industries; for example, they act as additives to inhibit lipid oxidation (19, 20). Lipid oxidation is responsible for unpleasant flavors and odors in food, making them unsuitable for consumption. To extend the shelf life of food products, companies frequently use synthetic antioxidants, such as butylated hydroxyanisole (BHA), a chief additive in the food industry (21). Although synthetic antioxidants are generally considered nontoxic, their use has some issues, as toxic effects have been detected in vitro and in vivo studies (22-24). It is proven that BHA can activate endocrine disorders and is also associated with liver cancers (25-27). Other studies have recognized the presence of synthetic phenol antioxidants in humans, such as in adipose tissues, serum, and urine (28, 29). Based on these facts, the use of synthetic antioxidants in foods is strictly limited in different countries. To overcome these problems, it is essential to find natural antioxidant alternatives that have low toxicity for human cells. These alternatives should be non-toxic and possess high antioxidant properties, allowing them to replace synthetic antioxidants.

*Cichorium intybus* from the Asteraceae family consists of several secondary metabolites, with main distribution regions in Europe and Asia. Various preparations of *C. intybus* are used to treat several illnesses. The literature review demonstrates that *C. intybus* L., the most well-known species of the *Cichorium* genus, has shown a wide range of ethnobotanical uses and has been widely employed for culinary purposes (30).

## 2. Objectives

The present study explores the antioxidant and cytotoxicity effects of biosynthesized AuNPs against liver cancer cells. The present work describes an easy method to fabricate AuNPs using *C. intybus* extract as eco-friendly, inexpensive stabilizing, and reducing agents. It also characterizes them using fourier-transform infrared spectroscopy (FTIR), field emission scanning electron microscopy (FESEM), and x-ray diffraction (XRD).

## 3. Methods

### 3.1. Materials and Reagents

All materials with analytical grade were purchased from Sigma-Aldrich, USA, for chemicals and were used as precursors for the preparation of AuNPs. The plant materials were collected from Kashmar (located in the northeast of Iran) on 10 May 2024.

### 3.2. Preparation of Herbal Extract and Gold Nanoparticle Synthesis

Five grams of powdered leaves were added to 100 mL of deionized water and stirred continuously for 24 hours at room temperature to ensure thorough extraction of the bioactive compounds. The resulting solution was filtered using Whatman filter paper to remove any particulate matter. The clear filtrate was stored in an Erlenmeyer flask and kept at 3°C to preserve its bioactivity for subsequent nanoparticle synthesis.

For the synthesis of AuNPs, a stock solution was prepared by dissolving 10 mg of HAuCl_4_ in 10 mL of deionized water. Subsequently, 10 mL of this stock solution was added to 90 mL of the herbal extract, resulting in a final volume of 100 mL. The mixture was shaken regularly at room temperature for 4 hours to facilitate the reduction of Au^3+^ ions and the formation of AuNPs. The synthesized AuNPs were then centrifuged at 10,000 rpm for 10 minutes to separate the nanoparticles from the reaction mixture. The supernatant was discarded, and the pellet containing AuNPs was washed once with distilled water to remove any unreacted components and impurities. This washing step was repeated to ensure the purity of the nanoparticles. The washed nanoparticles were then dried in a vacuum oven at 40°C for 24 hours to obtain powdered AuNPs. The dried nanoparticles were stored in an airtight container to prevent any contamination or oxidation until further use.

The green-fabricated AuNPs samples were exposed to various instrumental procedures such as FESEM (MIRA3, TESCAN), XRD (XRD Theta/Theta, Explorer, Italy), and FTIR (AVATAR 370 FT-IR, Thermo Nicolet, USA) analysis. Powdered AuNPs (obtained after washing) were used for characterization. For absorption spectra, FTIR, and FESEM analysis, powdered AuNPs were used to obtain their corresponding data.

### 3.3. DPPH Radical Scavenging Test

The antioxidant capacity of the AuNPs was assessed using the DPPH test, with BHA serving as a positive control. The free radical scavenging activity of AuNPs and standard BHA was determined using the stable radical DPPH. One milliliter of different concentrations (0, 125, 250, 500, and 1000 μg/mL) of AuNPs was mixed with 1 mL of freshly prepared DPPH (1 mM in ethanol) solution and vortexed thoroughly. The solution was then incubated at 25°C in the dark for 30 minutes. The absorbance was recorded at 517 nm using a spectrophotometer. The free radical scavenging activity was expressed as the percentage of inhibition, determined using the following formula:


$$\left(\%\right)\;of\;inhibition=({Absorbance}_{Control}\;-{Absorbance}_{Test}\;)/({Absorbance\;}_{Control})\times 100$$

### 3.4. Cytotoxicity of Gold Nanoparticles

The cell toxicity of the AuNPs was examined using the diphenyltetrazolium bromide (MTT) assay, a colorimetric method for assessing cell metabolic activity (31). Cell development was conducted in RPMI and DMEM media, supplemented with 100 μg/mL penicillin and 10% FBS, and then transferred to the incubator. Briefly, HepG2 cells were seeded in a 96-well plate and incubated for 24 hours. Various doses (0, 15.6, 31.2, 62.5, 125, and 250 μg/mL) of green-fabricated NPs were added. During this time, after each day of incubation, 20 μL of MTT dissolved in phosphate-buffered saline (PBS) was added to each well. At the end of the stage, the media was discarded, and the formed formazan crystals were dissolved in dimethyl sulfoxide (DMSO). Subsequently, the plates were shaken, and the absorbance was measured at 590 nm.

### 3.5. Statistical Analysis

The results of the MTT test, antioxidant activity, and significant differences were analyzed using SPSS software and one-way ANOVA with a statistical cut-off of P < 0.05. Graphs were drawn using Microsoft Excel software. The findings were presented as the mean ± SD, with three replications performed for each test.

## 4. Results

### 4.1. X-ray Diffraction Results in Biosynthesized Gold Nanoparticle

**Figure 1.**
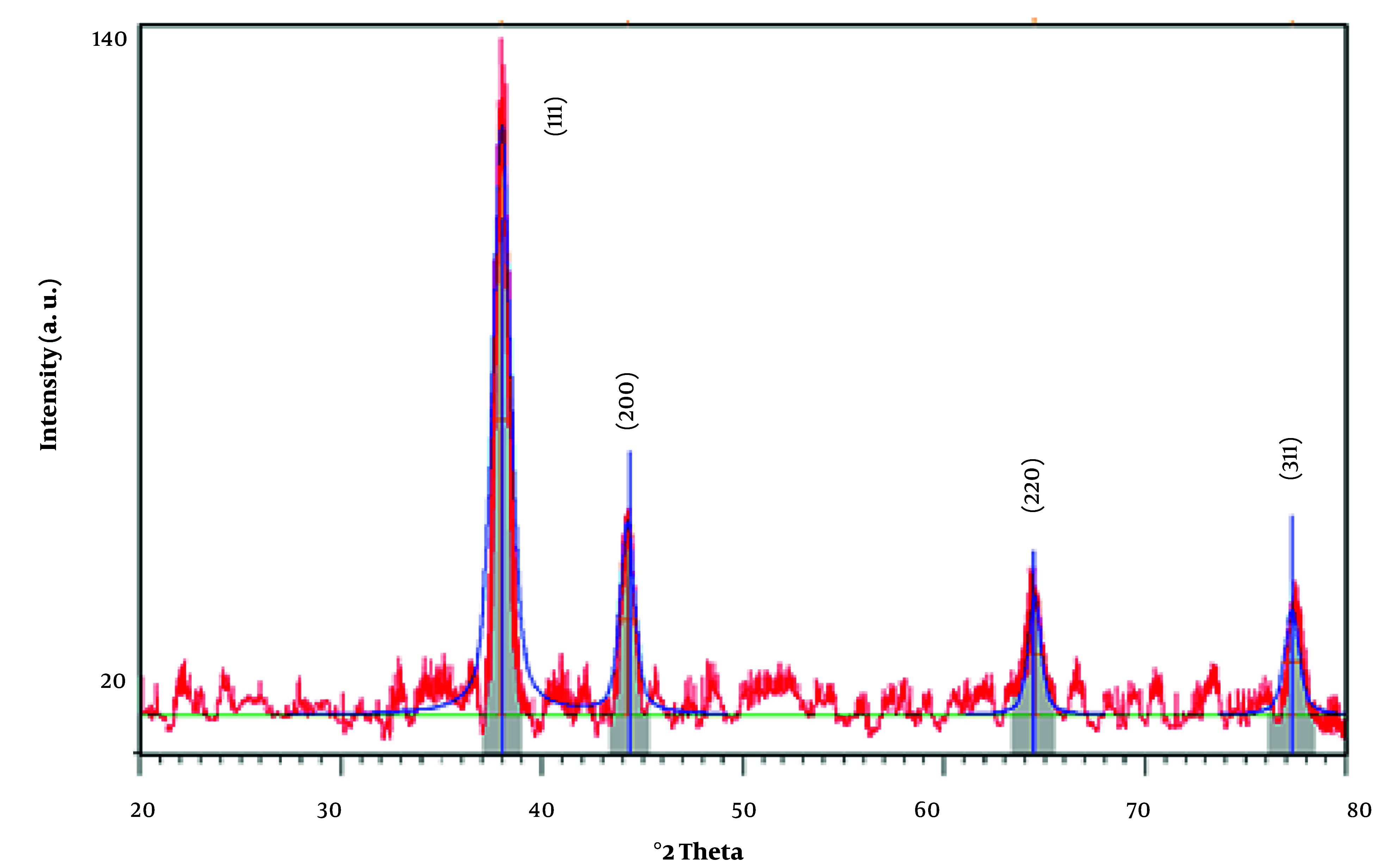
The PXRD analysis of the synthesized gold nanoparticles (AuNPs)

The XRD analysis of the synthesized AuNPs reveals a highly crystalline structure, consistent with the reference pattern for gold (Au) with a cubic crystal system (Figure 1). The diffraction peaks observed at 2Theta values of 37.9883°, 44.2480°, 64.4947°, and 77.3025° correspond to the (111), (200), (220), and (311) planes, respectively. These peaks align closely with the standard reference data (PDF code: 00-001-1172) (32), indicating the presence of face-centered cubic (fcc) gold.

### 4.2. Fourier-Transform Infrared Results and Interpretation

**Figure 2.**
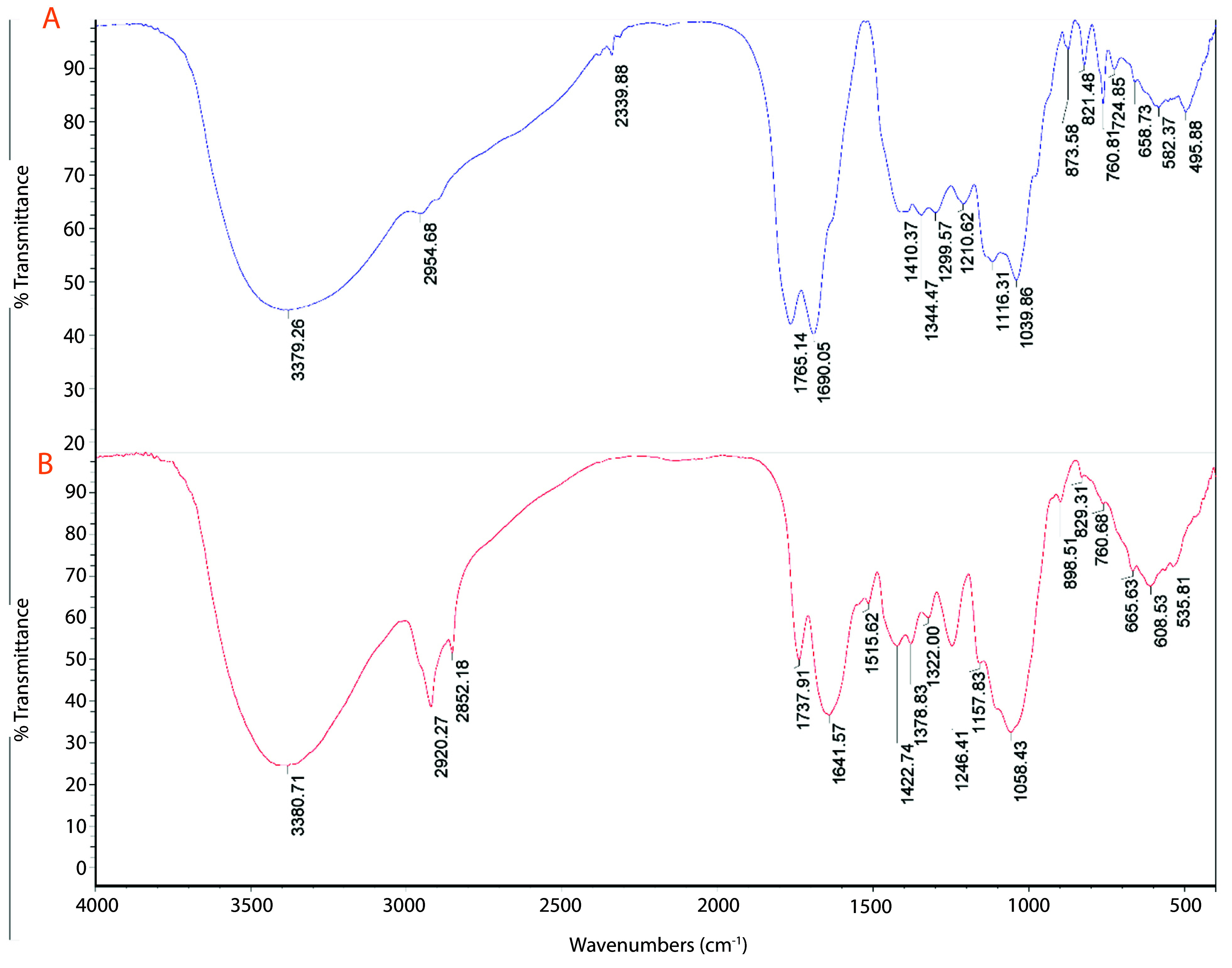
Fourier-transform infrared spectroscopy (FTIR) spectra of A, the plant extract; and B, biosynthesized gold nanoparticles (AuNPs). The spectra display characteristic peaks corresponding to various functional groups.

The FTIR spectrum of the plant extract exhibits characteristic peaks at various wavenumbers, indicating the presence of multiple functional groups (Figure 2). Notable peaks include those at 535.81 cm^-1^, 608.53 cm^-1^, and 665.63 cm^-1^, which are likely associated with C-H bending vibrations (33). Peaks at 760.68 cm^-1^ and 829.31 cm^-1^ correspond to aromatic C-H bending, while the peaks at 1058.43 cm^-1^ and 1157.83 cm^-1^ are indicative of C-O stretching and C-O-C stretching vibrations, respectively (34). The presence of peaks at 1246.41 cm^-1^ and 1322.00 cm^-1^ suggests C-N stretching and C-H bending vibrations. Additionally, the peaks at 1641.57 cm^-1^ and 1737.91 cm^-1^ correspond to the amide I band (C=O stretching) and carbonyl stretching vibrations, respectively (35). The broad peak at 3380.71 cm^-1^ is attributed to O-H stretching vibrations, indicating the presence of hydroxyl groups (36).

### 4.3. Electronic Microscopy and Spectrophotometric Analysis Results of Biosynthesized Gold Nanoparticle

**Figure 3.**
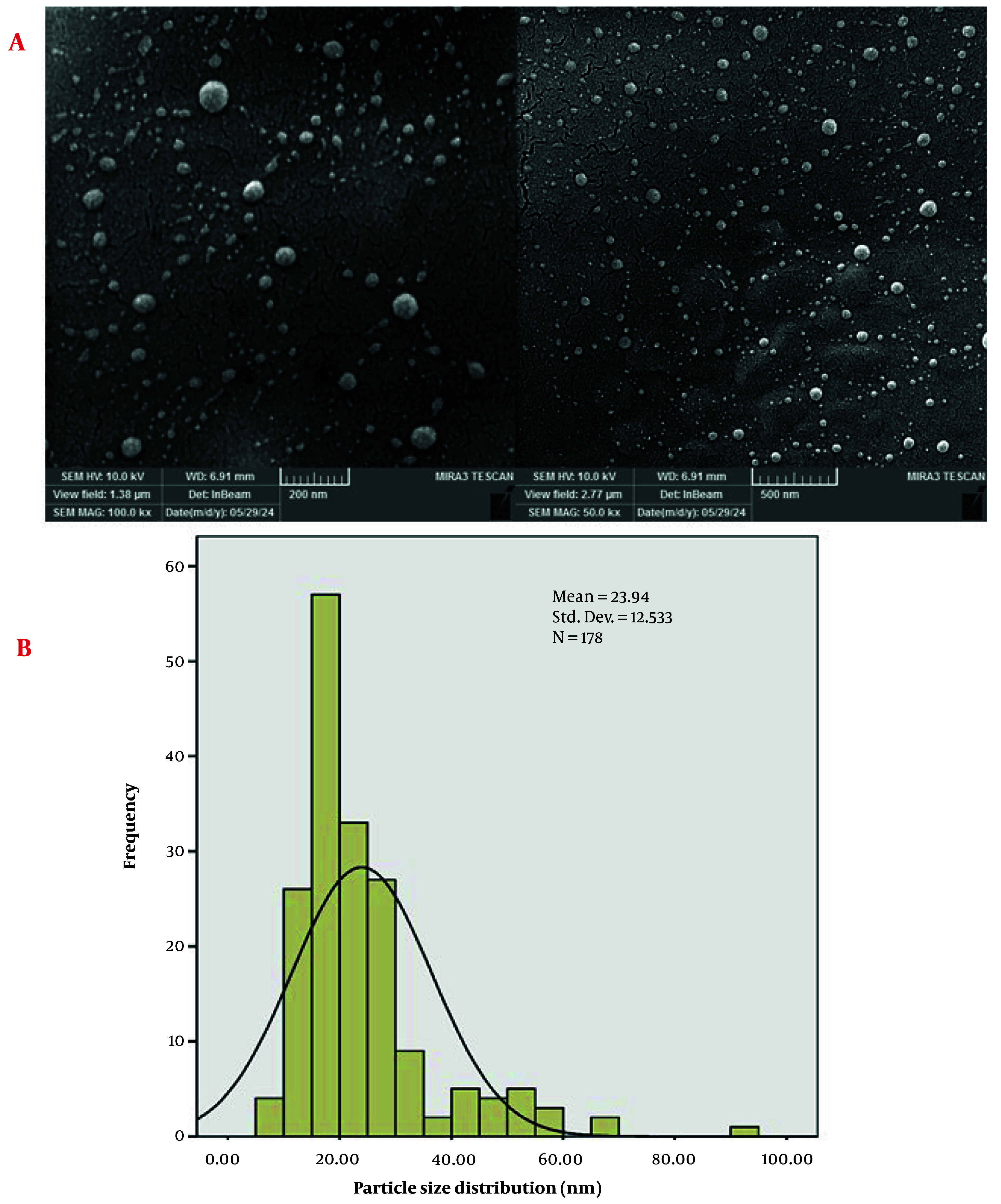
A, Field emission scanning electron microscopy (FESEM) images of gold nanoparticles (AuNPs) show the spherical morphology and smooth surface of the AuNPs; B, particle size distribution (PSD) of AuNPs

The FESEM images of the AuNPs provide detailed insights into their morphology and distribution (Figure 3). The high-magnification image on the left, with a scale of 200 nm, reveals that the AuNPs are predominantly spherical. The particles appear to be well-dispersed with minimal agglomeration, indicating a uniform synthesis process. The surface of the nanoparticles is smooth, which is characteristic of high-purity AuNPs.

The particle size distribution (PSD) of the AuNPs was analyzed using FESEM images, with measurements conducted using ImageJ software and statistical analysis performed with SPSS software (Figure 3). The histogram overlaid with a bell curve represents the frequency of particles within specific size ranges, indicating a normal distribution of particle sizes. The mean particle size of approximately 23.94 nm suggests that the majority of the AuNPs are within the nanoscale range, consistent with the high-resolution FESEM images. The standard deviation of 12.533 nm indicates some variability in particle sizes, which is typical for nanoparticles synthesized through green synthesis methods (37-39). This variability can be attributed to factors such as the dose of the reducing agent, reaction time, and temperature during the synthesis process.

The PSD analysis reveals that the AuNPs exhibit a relatively narrow size distribution, with most particles falling within the range of 10 to 40 nm. The bell curve suggests a normal distribution, indicating that the synthesis method produces nanoparticles with consistent sizes. The presence of a few larger particles, as indicated by the tail of the distribution, may be due to occasional agglomeration or variations in the synthesis conditions. Hence, the PSD analysis, combined with the FESEM images, confirms the successful synthesis of AuNPs with a mean size of 23.94 nm and a relatively narrow size distribution. This consistency in particle size enhances the potential applicability of the AuNPs in various fields, including catalysis, electronics, and biomedicine (40-43).

The difference between the PSD mean particle size (23.94 nm) and the crystallite size (8.31 nm) suggests that each gold nanoparticle is likely composed of multiple crystallites (44, 45). This is a common observation in nanomaterials, where individual nanoparticles can consist of several smaller crystalline regions. The larger particle size from the PSD analysis also accounts for any surface coatings or organic residues from the plant extract, which are not considered in the crystallite size calculation (46, 47).

The lower magnification image on the right, with a scale of 500 nm, provides a broader view of the nanoparticle distribution across the surface. This image shows that the AuNPs are evenly distributed, forming a relatively homogeneous layer. The absence of large aggregates suggests that the synthesis method effectively prevents particle agglomeration, which is crucial for maintaining the exclusive activity of nanoparticles (48, 49). The uniform distribution and spherical morphology of the AuNPs are essential for their potential applications (50-52). Therefore, the FESEM images confirm the successful synthesis of high-quality AuNPs with desirable morphological characteristics.

**Figure 4.**
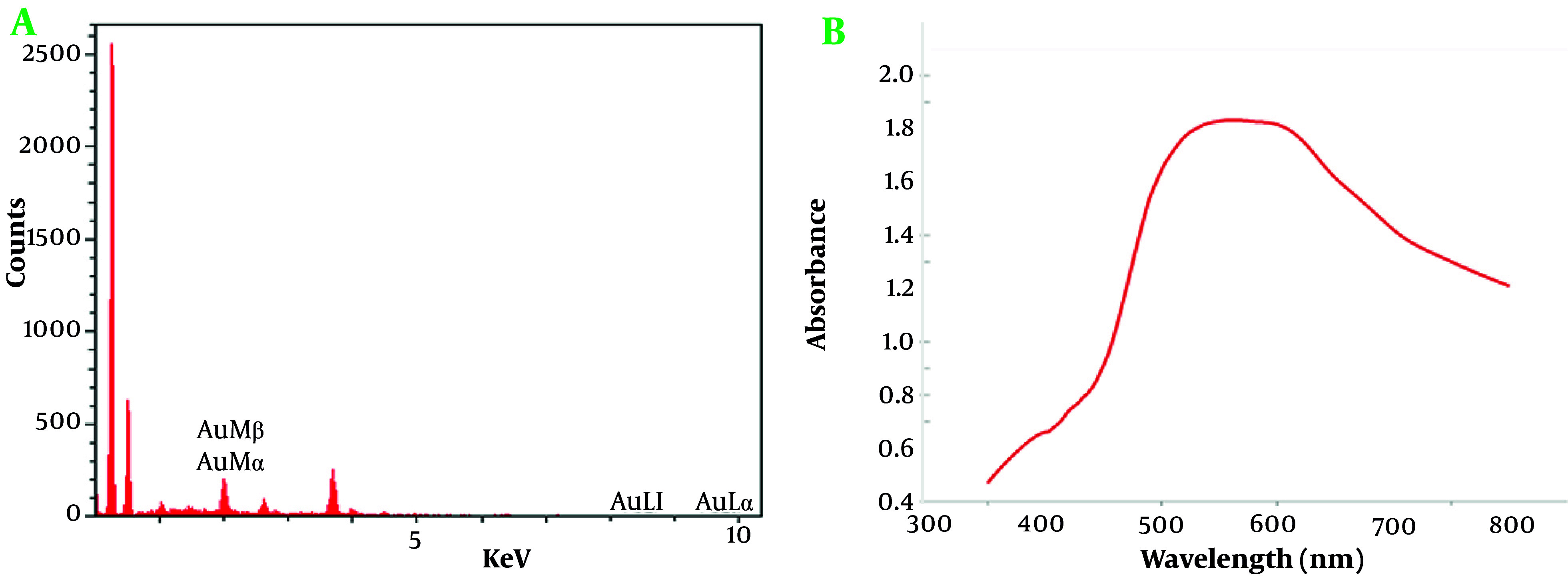
A, Energy-dispersive x-ray spectroscopy (EDX) spectrum of gold nanoparticles (AuNPs). The spectrum confirms the presence and high purity of gold in the nanoparticles; B, UV/Vis absorption spectrum of AuNPs

The energy-dispersive x-ray spectroscopy (EDX) analysis (Figure 4) conducted on the AuNPs reveals critical details about their elemental composition, confirming the high purity and concentration of gold within the sample. The EDX spectrum exhibits distinct energy peaks specifically associated with gold, including Au Mβ, Au Mα, Au Ll, and Au La lines. These characteristic peaks, unique to gold, substantiate the successful synthesis of AuNPs with a notable degree of purity, an essential requirement for advanced nanotechnology applications. The Au La line displayed an intensity of 3.6 with a relative error of 1.48%, signifying a robust signal that supports the presence of gold with minimal background interference. Gold was quantified at 100% by both weight and atomic percent, with no significant detection of other elements, validating the high purity of the AuNPs. The PAP (Phi-Rho-Z) correction was applied, enhancing the reliability of the data by compensating for any potential matrix effects.

The EDX analysis included a preliminary scan for other potential elements to confirm the purity of the sample. Elements such as iron (Fe), tantalum (Ta), iridium (Ir), dysprosium (Dy), rhenium (Re), and osmium (Os) were flagged with a 100% detection probability in general identification algorithms. However, these elements were absent from the actual sample spectrum, with no peaks of significant intensity, reinforcing the conclusion that the AuNPs were devoid of notable impurities.

In this analysis, an accelerating voltage of 15.0 kV was used to generate definitive energy peaks in EDX spectroscopy, enhancing elemental analysis reliability. A beam current of 10,000 nA provided a high signal-to-noise ratio for precise gold peak detection with minimal background interference. The analysis at 35,000x magnification allowed detailed visualization of the nanoparticle composition. Both live and preset times were 10 seconds, optimizing high-resolution spectra capture while minimizing noise. The detector, equipped with a 0.1 µm dead layer, minimized interferences for accurate low-energy x-ray detection. A 20 µm gold layer on the detector enhanced sensitivity to gold emissions, and a 10 mm^2^ active area provided clear spectral results. The silicon detector crystal, 3.0 µm thick with a 15 nm aluminum coating, was ideal for detecting x-rays across gold’s energy range.

The UV/Vis absorption spectrum of AuNPs provides valuable information about their optical properties and size distribution. The spectrum displays a distinct peak in the absorbance curve around the 520 - 540 nm range, which is characteristic of the surface plasmon resonance (SPR) of AuNPs (Figure 4). This SPR peak arises due to the collective oscillation of conduction electrons on the nanoparticle surface when exposed to light, and it is a key feature of metallic nanoparticles (53).

### 4.4. Antioxidant Activity Results of Gold Nanoparticle

**Figure 5.**
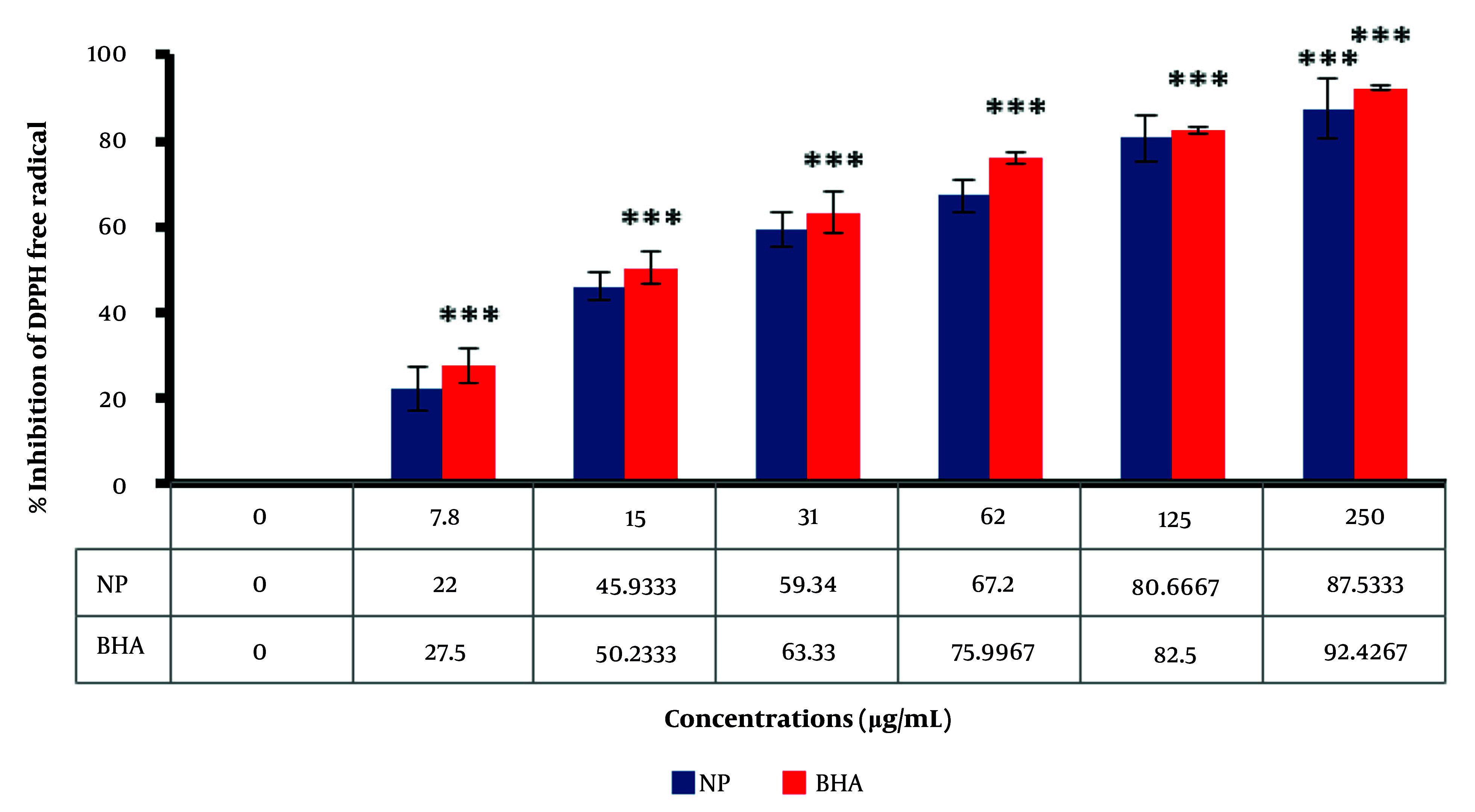
The inhibitory result of the fabricated gold nanoparticles (AuNPs) on DPPH free radicals compared to butylated hydroxyanisole (BHA) as a positive control. Asterisk symbols show a significant difference between experimental groups (*** P < 0.001).

The DPPH radical is widely used in evaluating free radical scavenging activity due to the simplicity of the reaction (54). The absorption properties of the DPPH radical demonstrate the effectiveness of the green-fabricated AuNPs formed from the extract, compared with BHA as a positive control. As shown in Figure 5, AuNPs can scavenge DPPH radicals in a dose-dependent manner, with antioxidant activity increasing as the dose of nanoparticles is enhanced. Moreover, the results indicate that AuNPs are more effective than BHA, an industrial antioxidant, suggesting that AuNPs have greater potential than industrial antioxidants. By increasing the concentration of biosynthesized AuNPs, the percentage inhibition of DPPH free radicals increases. At a concentration of 250 µg/mL, the percentage inhibition of DPPH free radicals for biosynthesized AuNPs and BHA was observed to be 87.5% and 92.4%, respectively.

### 4.5. Cytotoxicity Results of Gold Nanoparticles Against Cancer Cell Line (HepG2)

**Figure 6.**
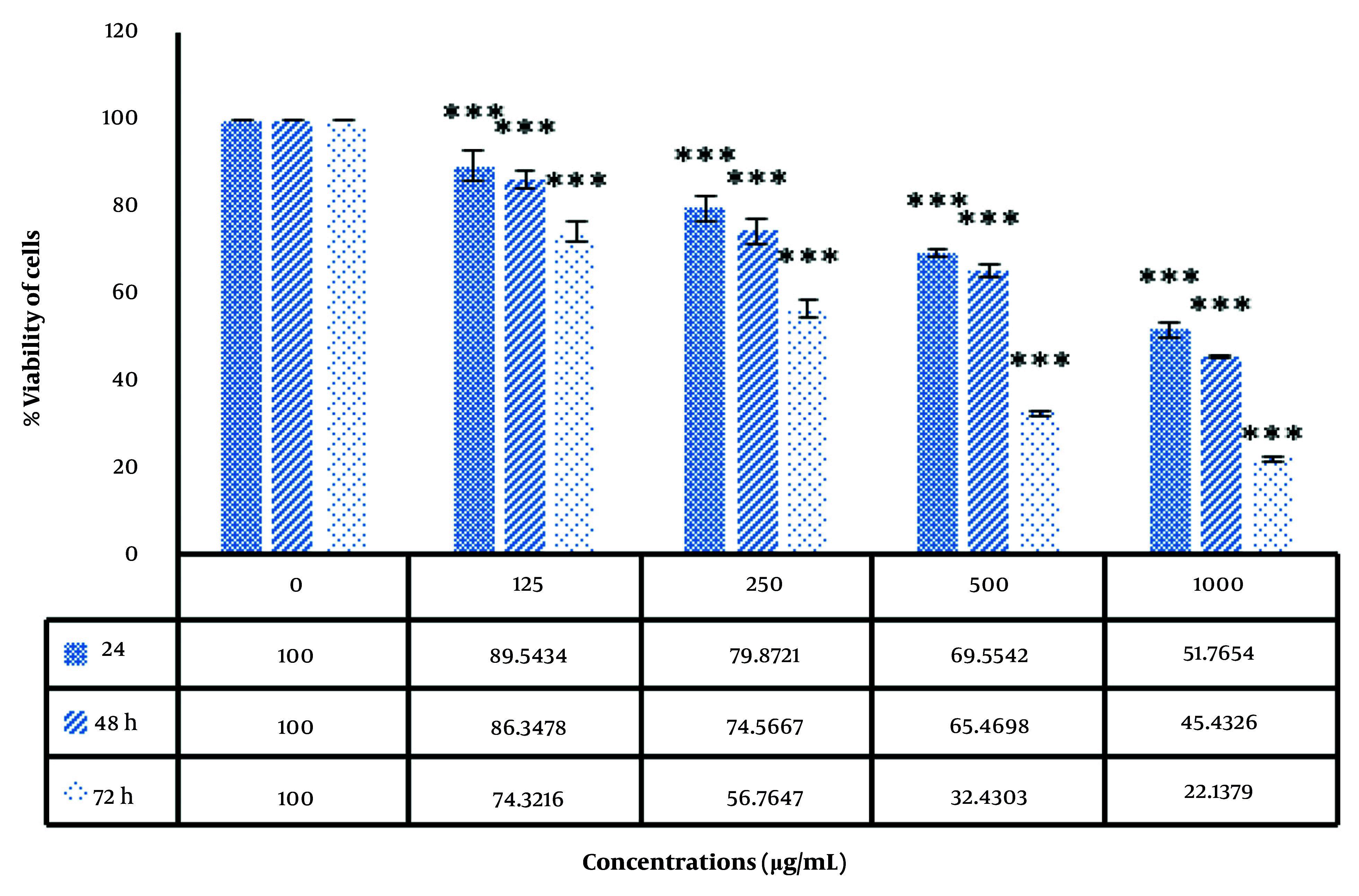
Cytotoxicity effect of gold nanoparticles (AuNPs) on cancer cell line (HepG2). Asterisk symbols show a significant difference between experimental groups (*** P < 0.001).

As displayed in Figure 6, the induction of cellular toxicity by the biofabricated AuNPs is dependent on both the dose and duration of treatment. As the concentration of nanoparticles increases, the death rate of cancer cells also rises.

## 5. Discussion

The comparison of the FTIR spectra of the AuNPs and the plant extract indicates that several functional groups from the plant extract are present on the surface of the AuNPs. Peaks such as those at 760.81 cm^-1^, 1641.57 cm^-1^, and 1737.91 cm^-1^ in the plant extract are also observed in the AuNPs spectrum, suggesting that these groups are involved in the reduction and stabilization of the AuNPs. The presence of new peaks at 495.88 cm^-1^ and 2339.88 cm^-1^ in the AuNPs spectrum may indicate interactions between gold and organic molecules from the plant extract, which are crucial for the formation and stabilization of the nanoparticles (55-59). Overall, the FTIR analysis confirms that the green synthesis method effectively incorporates functional groups from the plant extract onto the surface of the AuNPs, contributing to their stability and potential applications in various fields.

Li et al. examined the antioxidant properties of flavonols, AuNPs, and flavonol-AuNPs using the DPPH method. Their results showed that flavonol-AuNPs exhibited significantly higher antioxidant properties compared to both flavonol and AuNPs when examined separately (60). Another study examined the antioxidant properties of AuNPs fabricated by *Spatoglossum asperum*. The results showed that synthesized AuNPs, with a 20 nm diameter, significantly scavenged the DPPH free radical, showing 73.21% inhibition at a dose of 50 µg/mL (61).

Biosynthesized AuNPs were observed to induce cytotoxicity in HepG2 cells, and the results were consistent with other reports stating that synthesized AuNPs induced oxidative stress in a dose-dependent manner, as evidenced by a decline in ROS creation and lipid peroxidation (62, 63). The cell toxicity mechanism of AuNPs generally involves the over-generation of ROS, which mediates apoptosis, causes upregulation of the BAX gene, and activates the caspase cascade while suppressing Bcl_2_ (64-66).

Table 1 shows the antioxidant and cytotoxicity properties of some investigations of green synthesized AuNPs and their properties. However, physicochemical procedures are extensively employed for the large-scale production of AuNPs to meet their exponentially growing potential uses in biomedical fields. The use of unsafe and expensive materials poses a significant challenge in the production of nanoparticles (67). Consequently, the challenges associated with conventional fabrication methods have motivated researchers to explore green, safe, and cost-effective approaches for the fabrication of AuNPs to meet the growing industrial demand (68-70). The photo-fabrication of AuNPs is recognized as an important method due to its eco-friendly and renewable properties. However, the use of commercially valuable herbs as reducing agents affects the effectiveness of the fabrication process. Therefore, there is a pressing need to shift focus towards discovering the reduction potential of herbal-mediated wastes for the fabrication of AuNPs. While numerous studies have confirmed the promising biocompatibility of AuNPs, comprehensive toxicological in vitro and in vivo assays are critical before AuNP-based treatments can be safely employed in humans.

**Table 1.** Comparison of Some Studies of Plant-Mediated Synthesized Gold Nanoparticles

Source	Size (nm)	Morphology	Findings	Reference
***Martynia annua***	21	Spherical	The AuNPs exhibit robust DPPH scavenging activity and effective reducing power; the AuNPs exhibit robust DPPH scavenging activity and effective reducing power.	(71)
***Allium cepa***	6 - 54	Spherical	The AuNPs had lower activity when compared to normal antioxidants.	(72)
***Nepeta bodega***	20.5	Spherical	The AuNPs exhibited concentration-dependent cytotoxicity on the human liver (HepG2) and breast (MCF-7) cancer cells, which were around 77.4 and 71.2% at 300 µg/mL, respectively.	(73)
***Pandanus canaranus***	37.4	Spherical	As the concentration of AuNPs increased, the findings of cell viability reduced in the lung cancer cells (A549 cells).	(74)
***Ginkgo biloba***	18	Spherical	The AuNPs exhibited promising in vitro dose and time-dependent antiproliferative effects against human nasopharyngeal carcinoma cells.	(75)
***Coleus scutellarioides***	40	Spherical	A concentration-dependent impact of AuNPs on human breast cancer cells.	(76)
***Curcuma longa***	10 - 15	Semi-spherical	The AuNPs had more antioxidant activity when compared to BHA; the % lung cancer cell viability decreased significantly in all of them as the AuNPs dose increased.	(77)
***Thespesia lampas***	43	Spherical	The AuNPs were significantly effective (50.47%) against head and neck cancer cells at a higher dose of 100 μg mL^-1^; the NPs had significant antioxidant activity when compared to standard antioxidants.	(78)
***Cucurbita moschata***	≤ 100	Spherical	The AuNPs exhibited better antiproliferative activity with 47.19% viability in lung cancer cells and 42.76% in human ovarian cancer cells at a 50 μg/mL concentration.	(79)
**Papaya**	44.4	Spherical	The AuNPs displayed a significant antioxidant effect (93.24% DPPH scavenging and 74.23% SOD inhibition at 100 µg/mL.	(80)

Abbreviations: AuNPs, gold nanoparticles; BHA, butylated hydroxyanisole.

### 5.1. Conclusions

In this work, AuNPs were fabricated using an extract of *C. intybus*. The synthesized nanoparticles were fully characterized by FESEM, PXRD, and FTIR, with a diameter size of 23.94 nm. It appears that the extract of *C. intybus* prevented the aggregation of AuNPs. The extract is primarily responsible for the fabrication of AuNPs, and the nanoparticles demonstrated significant cellular toxicity effects against cancer cells in a time- and concentration-dependent manner. Additionally, antioxidant experiments indicate that these nanoparticles can be employed as alternatives to synthetic antioxidants. The simplicity, low cost, and effectiveness of this procedure suggest an alternative to conventional synthetic approaches for AuNPs. The environmentally friendly fabricated AuNPs can be valuable and effective in the pharmacology and food industries.

## Data Availability

The dataset presented in the study is available on request from the corresponding author.
